# Iceberg of workplace violence in health sector of Bangladesh

**DOI:** 10.1186/s13104-018-3795-6

**Published:** 2018-10-04

**Authors:** Md Imran Hasan, Md Zakiul Hassan, Md Mofijul Islam Bulbul, Taufique Joarder, Mohammod Jobayer Chisti

**Affiliations:** 1 Mymensingh Medical College, University of Dhaka, Dhaka, Bangladesh; 2 Sir Salimullah Medical College, University of Dhaka, Dhaka, Bangladesh; 3 National Nutrition Services, Ministry of Health and Family Welfare, Dhaka, Bangladesh; 4 Department of International Health (Health Systems), Johns Hopkins Bloomberg School of Public Health, Baltimore, MD USA; 5 FHI 360, Dhaka, Bangladesh; 6 The University of Melbourne, Victoria, Australia

**Keywords:** Iceberg, Workplace violence, Health sector, Healthcare worker, Bangladesh

## Abstract

**Objectives:**

‘Negligence of Physicians’ and ‘Wrong Treatment’ have become commonly-used phrases in print and electronic media of Bangladesh, while violence against healthcare workers has always been under-reported. Unfortunately, there is little evidence regarding physical violence against healthcare workers, while there is no data on the magnitude of psychological violence. The objective of this study was to quantify and explore the magnitude of workplace violence in health sector of Bangladesh to guide future research and adopt preventive policies.

**Results:**

The Majority (96%, n = 54) of the violence cases were physical in nature and 91% violence (n = 51) took place in public healthcare settings. More than one-third (39%) of the violence cases occurred at primary healthcare level and one-third (39%) at tertiary healthcare level. It was mostly (61%) the entry-level physicians who were affected by violence. The report reveals the tip of the iceberg of workplace violence in health sector of Bangladesh. Further studies should be undertaken to assess the prevalence, magnitude, and associated factors for workplace violence against healthcare workers.

## Introduction

Workplace violence, either physical or psychological, has become a global problem crossing geographical borders, work settings, and occupational groups. While workplace violence affects practically all sectors and all categories of workers, the health sector is at major risk. Violence in this sector may constitute almost a quarter of all violence at work all over the world [1].

Under the eclipse of reforms, growing work pressure and stress, social instability and the deterioration of inter-personal relationships, workplace violence is rapidly spreading in the health sector. Recent studies confirm that workplace violence in the health sector is universal, although local characteristics may differ. Altogether it may affect more than half of the healthcare workers [2].

Workplace violence is prevalent in both developed and developing countries. In 2011, a national survey in the United States reported that 78% of the emergency medicine residents and attending physicians had experienced violence at the workplace [3]. In 1999, a study in Canada found that 68% of physicians reported an increased frequency of violence over time, and 60% reported an increased severity. 76% of the respondents witnessed verbal abuse, 86% witnessed physical threats or assaults and 57% were physically assaulted [4]. Researchers investigating workplace violence from European, Asian and Middle Eastern countries found a high prevalence of physical and psychological violence in the health sector of those countries [5–11].

**Fig. 1:**
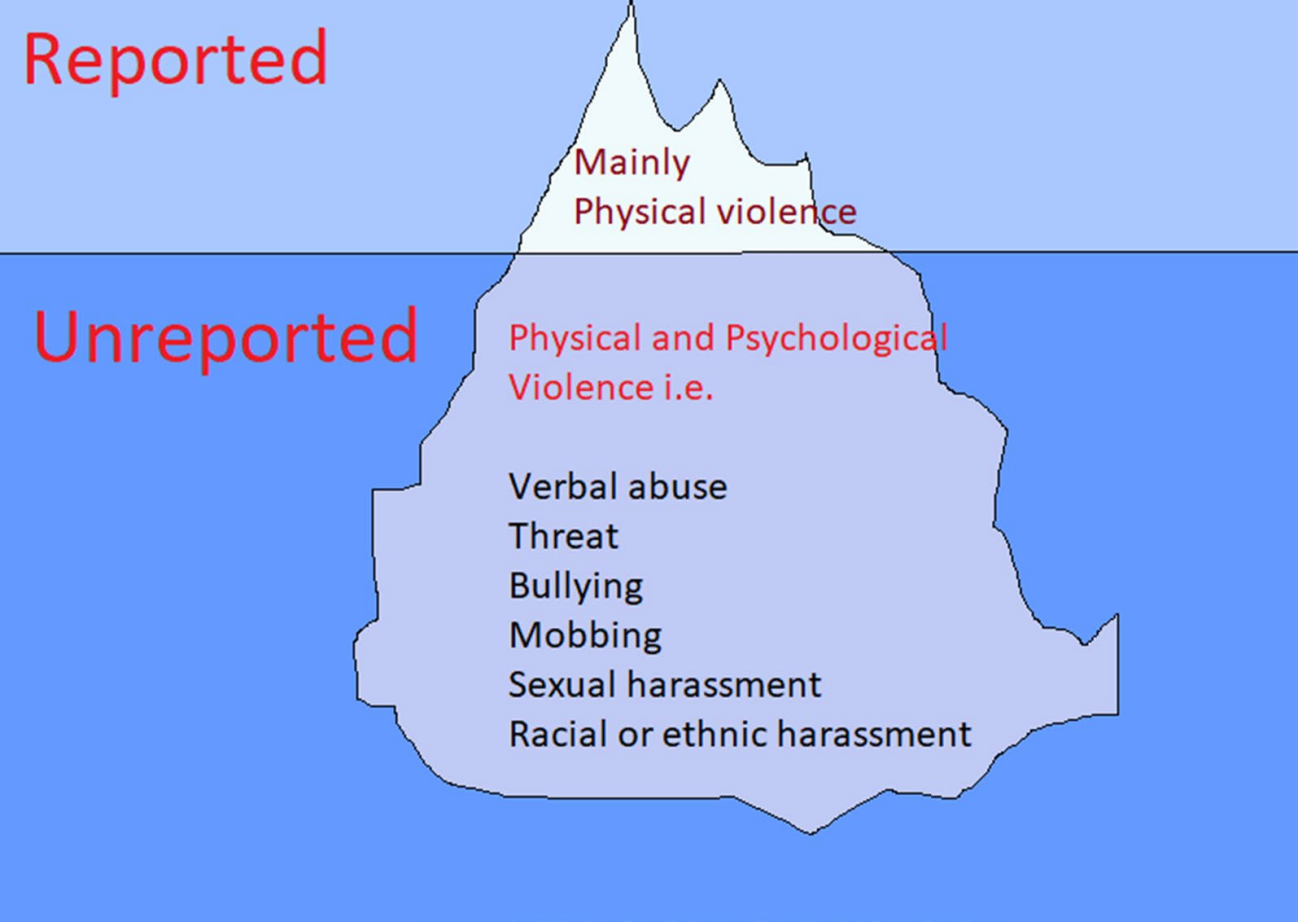
Conceptual framework of iceberg of workplace violence in the health sector

Although healthcare providers are increasingly concerned about the escalating incidents of workplace violence, there is a lack of evidence to support this concern due to low reporting rates. A study found that only around 15% of workplace violence cases were reported to police or public security authorities [12]. In addition, sometimes these cases were reported as ‘Negligence of Physicians’ without proper investigation by the concerned authorities. The reported cases of violence form the tip of the iceberg while non-reported cases of violence remain as the submerged portion of the iceberg. Figure 1 shows conceptual framework of reported and not reported cases of workplace violence.

The negative consequences of such widespread violence affect heavily on the delivery of healthcare services, calling upon the quality of care. Besides, this is provoking the decision of the health workers to leave their professions. This, in turn, can result in a reduction in health services available to the general population, and an increase in health costs through defensive medical practice [1]. In developing countries like Bangladesh, equal access of care seekers to primary health care may be threatened if already scarce healthcare workers [13] abandon their profession because of the threat of violence [1].

Recently violence against the physician in Bangladesh has been increased and the severity has been intensified simultaneously. Along with emergency department, indoor departments also undergo violence either by patients or attendants of patients [14–17]. These eventually hamper the healthcare services for the innocent and poor patients.

Unfortunately, there is very limited data on violence against healthcare workers in Bangladesh. If we get an insight on the prevalence and associated factors of workplace violence, it will guide us to adopt preventive measures and workplace safety policies. The objective of this review was to explore the magnitude of workplace violence against healthcare workers to guide future research in identifying strategies that could effectively reduce the incidence of workplace violence in Bangladesh.

## Main text

### Methods

We surveyed an online newspaper named ‘Platform’ [18], which is published by a group of medical professionals that reports all news articles related to medical science and public health in Bangladesh. ‘Platform’ is run by “Platform Organization for Medical and Dental Society”, which is a non-profit, non-political, non-government voluntary organization for physicians, medical and dental students of Bangladesh. It endeavors to provide an open platform for all medical personnel of Bangladesh contributing to various professional and social causes and offers a forum for peer-to-peer information sharing. Consequently, any violence against medical professionals in any corner of the country gets shared in the forum instantly and published online before other news media reports it.

‘Platform’ publishes news reports after several confirmations including both official and unofficial confirmation, i.e., peer confirmation. Therefore, we considered all reported news to be authentic. We assessed all the news articles that were published between May 2014 and May 2017. Total 582 news articles were reviewed; among which 68 articles were identified for reporting violence against medical professionals. Multiple news for a single event was counted only once. Finally, 56 news articles were retrieved and incorporated into the database with the unique identification number in accordance with variables. The whole process was independently reviewed by two independent researchers and cross-matched subsequently. Contradictions between the two reviewers were solved by a third reviewer.

We identified variables from World Health Organization (WHO) guideline on workplace violence in health sector [19]. Identified variables were place of violence, type of healthcare setting, level of healthcare setting, department of healthcare setting, type of violence, professional category of the victim, immediate consequence of violence and follow up of violence. Two independent researchers conducted the content analysis of news articles and identified the selected variables. A third reviewer crosschecked and compiled the data and did descriptive analysis by using a statistical package SPSS, version 20.0.

Definitions and terms:

Classification of healthcare settings [20]:

- Primary: Sub-district level health centers (Upazilla Health Complex) and below, i.e. Union Sub-Centers and Community Clinics and equivalent private healthcare providers.
- Secondary: District level health centers (District Hospitals) and equivalent private healthcare providers.
- Tertiary: National Institutes, Medical Universities, Medical Colleges and equivalent private hospitals etc.

Classification of healthcare providers:

Most of the news articles reported violence against physicians, overlooking or underestimating violence against other healthcare workers. As physicians are directly related with treating patients and objected to violence, we only classified physicians according to existing hierarchy.

- Internship physician: Those who have passed final professional examination under a university and hold a provisional registration from Bangladesh Medical and Dental Council (BMDC).
- Entry-level physician: Those who work as House Officer or Medical Officer.
- Mid-level physician: Those who work as Assistant Registrar and Registrar.
- Consultant: Those who have completed post-graduation and work as Junior Consultant and Assistant Professor.
- Senior professional: Those who work as Senior Consultant or Associate Professor and above.

### Results

**Fig. 2:**
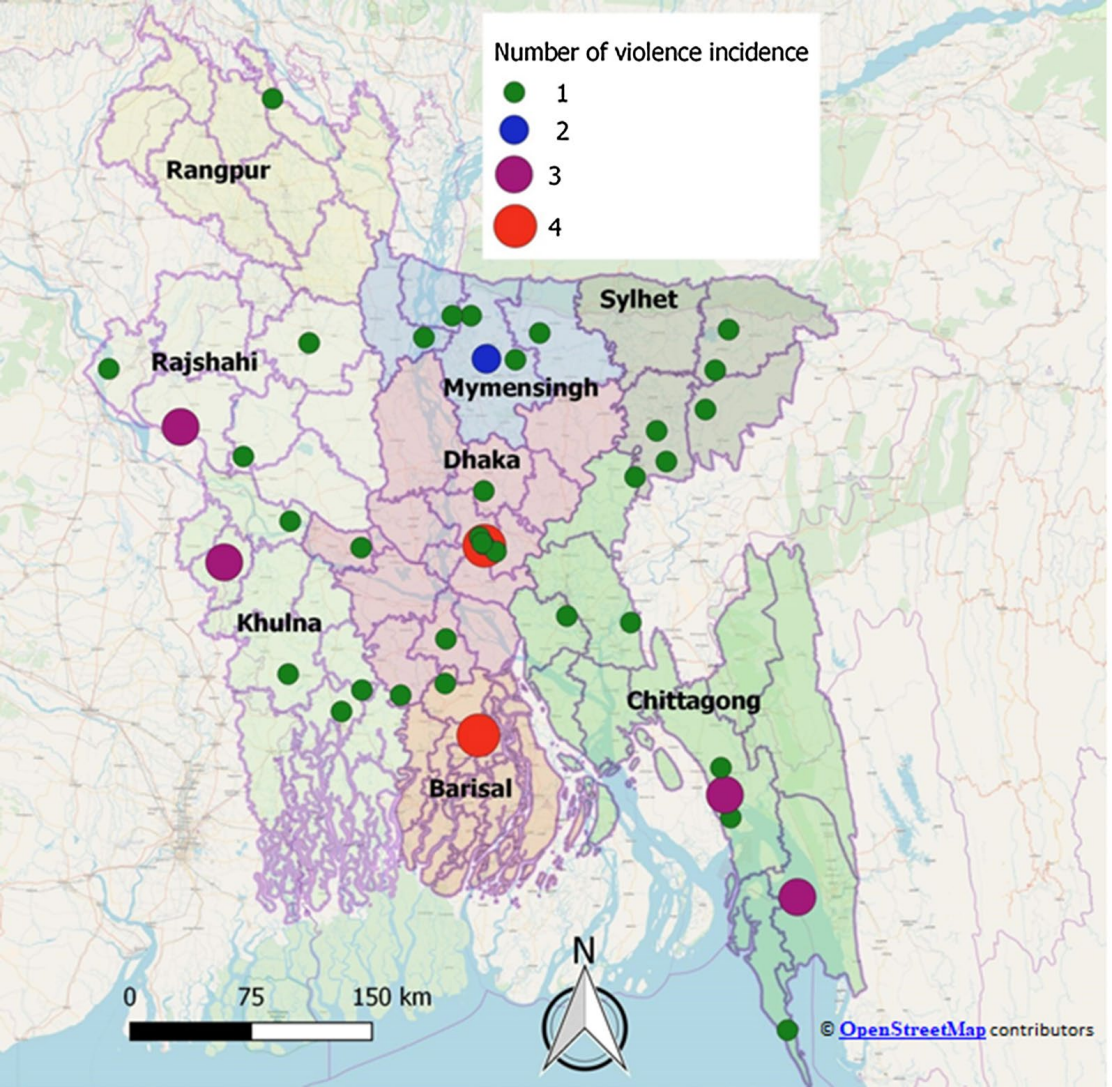
Distribution of workplace violence in different health facilities by sub-districts (upazilla) of Bangladesh during May 2014–May 2017

The review revealed that incidents of violence against healthcare workers were distributed throughout the country, which is illustrated in Fig. 2. Around 91% of violence took place at public healthcare centers. One-third of the violence (39%) occurred at primary healthcare facilities and one-third (39%) at tertiary healthcare facilities. Around 52% and 41% of violence were manifested at emergency and indoor department, respectively, while violence at the outdoor department was scarce. Of 56, 54 (96%) reported cases of violence were physical in nature and two reports mentioned of psychological violence. Most of the reports (n = 54) covered violence against physicians. Entry-level physicians were the most affected group (61%). One-third of violence led to strike and interruption of healthcare services. Most of the violence did not have a follow-up report or any update regarding intermediate and late consequences of that violence. Frequencies of workplace violence against healthcare workers in Bangladesh are summarized in Table 1.

**Table 1:** Different traits of reported workplace violence in health sector of Bangladesh between May 2014 and May, 2017

Different traits of violence	Frequency (%)
*Type of healthcare facility*	
Public	51 (91.1%)
Private	5 (8.9%)
*Level of healthcare facility*	
Primary	22 (39.3%)
Secondary	12 (21.4%)
Tertiary	22 (39.3%)
*Department of healthcare facility*	
Emergency	29 (51.8%)
Indoor	23 (41.1%)
Outdoor	2 (3.6%)
Other	2 (3.6%)
*Type of violence*	
Physical	54 (96.4%)
Psychological	2 (3.6%)
*Professional category of victim*	
Physician	54 (96.4%)
Nurse	1 (1.8%)
Other	1 (1.8%)
*Professional status of victim*	
Internship physician	9 (16.1%)
Entry level physician	34 (60.7%)
Mid-level physician	7 (12.5%)
Consultant	2 (3.7%)
Senior professional	2 (3.7%)
Other	2 (3.7%)
*Immediate consequence of violence*	
Strike	17 (30.4%)
Peaceful protest	15 (26.8%)
Stoppage of services	5 (8.9%)
Law enforcement	6 (10.7%)
Not reported	13 (23.2%)
*Follow up of violence*	
Yes	2 (3.6%)
No	54 (96.4%)

### Discussion

The study revealed that violence against health professionals was prevalent in all healthcare levels. No healthcare settings or professionals were immune to violence. All violence ended up in the strike, stoppage of services or other forms of health services distraction causing unbearable sufferings for the general service seekers, along with victims and offenders.

The study found 56 cases of violence within the three-year period. It did not necessarily represent all violence at healthcare centers, rather a small portion of violence instead. Only cases of physical violence were reported, in some cases, under-reported. However, psychological violence was hardly reported. In a study, Gerberich et al. found that only 15% of the violence incidences in the health sector were reported [12]. Lack of administrative support, lengthy legal procedures, and fear of injustice contribute to low reporting of violence incidents in health sector [21].

Majority of the violence took place in primary and tertiary healthcare settings. Critical patients from rural areas first present at the primary health centers. Some of these patients deteriorate or die at primary centers, which may end up in violence. A study in the US also reported that working in the community settings was associated with violence [22]. Another study in Serbia reported that workplace violence prevailed in primary healthcare centers [23].

As the health system of Bangladesh does not have a proper referral system, some of these critical patients get referred directly to tertiary centers bypassing the secondary healthcare facilities. Moreover, critical patients from urban and semi-urban areas visit tertiary the health centers directly. This practice causes huge workload on healthcare workers, and consultation time gets shortened. Eventually, it may lead to lack of patients’ satisfaction followed by violence at tertiary healthcare centers. A study in Greece also reported a high prevalence of workplace violence at the tertiary hospital [7].

Around half of the reported physical violence took place at emergency departments. As emergency is the door of indoor departments, emergency department followed by the indoor department first deals critical patients. Some of these critical patients die either at emergency or indoor ward even after attending physicians’ best efforts and these usually cause physical violence. Studies in different countries also emphasize that emergency departments are more vulnerable to experience physical violence [24–26].

This study showed that entry-level physicians were mostly affected by violence. Internship physicians and entry-level physicians are the preliminary and emergency service providers and patients get to see senior physicians after being stabilized. Therefore, interns and entry-level physicians are more prone to violence. Similarly, a study in the United States reported that age and years of experience were the risk factors for violence [22].

### Conclusions

This study unveils the tip of the iceberg of workplace violence and guides to many research questions, e.g., the prevalence, magnitude, and possible risk factors for workplace violence against healthcare workers in Bangladesh. Further studies should be undertaken to assess and examine the consequences of violence, professionals’ incident reporting patterns, and existing violence prevention and safety measures from healthcare workers’ perspectives. As psychological violence was not reported or under-reported, longitudinal studies should be undertaken to investigate the depth of psychological violence. Similarly, violence against nurses and other supporting health staffs needs further investigations. The government should adopt workplace safety policy in the health sector, facilitate, and promote health services and health policy research to find innovative and cost-effective ways to combat this situation.

## Limitations

There are several limitations in our review. First, despite our intention to conduct a comprehensive search for articles, it is possible for us still to miss some media reports on workplace violence on physicians. Second, we did not review the social media, e.g., Facebook, Twitter, etc., where there are extensive and informal discussions on workplace violence in the health sector. The breadth and volume of information on social media will definitely require a separate study and analysis.

## Abbreviations

BMDC: Bangladesh Medical and Dental Council
SPSS: Statistical Package for the Social Sciences
US: United States
WHO: World Health Organization
